# Industrial Wastewater Treatment by Nanofiltration—A Case Study on the Anodizing Industry

**DOI:** 10.3390/membranes10050085

**Published:** 2020-04-29

**Authors:** Aamer Ali, Maria C. Nymann, Morten L. Christensen, Cejna A. Quist-Jensen

**Affiliations:** Center for Membrane Technology, Department of Chemistry and Bioscience, Aalborg University, Fredrik Bajers Vej 7H, 9220 Aalborg East, Denmark; aa@bio.aau.dk (A.A.); maria.nymann@hotmail.com (M.C.N.); mlc@bio.aau.dk (M.L.C.)

**Keywords:** nanofiltration, anodizing, wastewater, heavy metals, water reuse, membranes

## Abstract

The anodizing industry generates several alkaline and acidic wastewater streams often with high concentrations of heavy metals. In this study, nanofiltration (NF) was used to treat wastewater from individual baths, i.e., wastewater from color rinse, alkaline pickling rinse, acidic pickling rinse and anodizing rinse, as well as a mixture of all the wastewater streams. The experiments were carried out by using a commercial membrane (NF99HF) exhibiting pure water permeability of 10 L/(m^2^·h·bar). For all wastewater streams except one, pH was adjusted to bring it within the recommended pH limits of the membrane, whereby part of the heavy metals precipitated and was removed. The NF of the color rinse offered high-quality permeate (heavy metals below detection limit) and high permeability (9 L/(m^2^·h·bar)), whereas the nanofiltration of the alkaline pickling rinse exhibited no permeability. The NF of the acidic pickling rinse showed a permeability of 3.1–4.1 L/(m^2^·h·bar), but low ion rejection (7–13%). NF of the neutralized mixed wastewater, after the removal of precipitate, produced high-quality permeate with a stable permeability of 1 L/(m^2^·h·bar). Treatment of the mixed wastewater is therefore the best option if the water has to be discharged. If the water has to be reused, the permeate conductivity in the color rinse and anodizing rinse baths have been reduced significantly, so the treatment of these streams may then be a better option.

## 1. Introduction

Anodizing is an important process to provide superior esthetic and anti-corrosion properties to various metallic surfaces. In addition to the main anodizing step, the overall process consists of several pre and post-treatments [1]. The main pretreatments include degreasing and pickling aimed at removing mainly grease and the metal oxide layer from the surface. Coloring and sealing are performed as the main post treatment steps with objective of coloring and closure of the surface pores, respectively [2]. After completing each step, aluminum parts are moved to rinsing baths to remove residuals. Thus, each rinsing step produces both alkaline and acidic wastewater streams, which contain heavy metals such as chromium, lead, zinc, copper and manganese, often in higher quantities than the permitted limits for discharge [3]. Heavy metals might accumulate in living organism and have serious impacts even when present in trace concentrations [4]. Thus, the discharge of these streams into the environment is a concern, meaning that proper treatments are needed before discharge [5].

Traditionally, the effluents from the anodizing industry are treated by operations involving neutralization, flocculation, settling and press filtration and the sludge is often disposed on land [6]. Generally, the acidic and alkaline streams are collected separately and fed to the treatment plant in a controlled flow for neutralization [7,8]. An additional neutralization step might be needed depending upon the degree of neutralization achieved after mixing acidic and alkaline streams in the treatment plant. Subsequently, the wastewater is added to a coagulation process followed by the settling. The sediments from the settling tank are introduced into a filter press to achieve a final solid concentration as high as 30% [7]. However, the process consumes large amounts of chemicals, generates secondary waste such as hydroxides and has large footprints [9,10]. Moreover, the final effluent still might contain heavy metals in trace quantities [11].

Membrane processes have gained significant attention as a replacement of traditional unit operations in desalination and wastewater treatment [12] due to their compact size, less energy intensive nature, efficient separation capabilities and environmental friendly nature due to less chemical consumption. Nanofiltration (NF) is a pressure-driven membrane process with separation efficiency between reverse osmosis and ultrafiltration. NF is generally carried out by using asymmetric polymeric membranes consisting of a functionally active porous top layer with a low resistance support layer. Typical pores in the active layer are around 1 nm in size and have fixed charges. Thus, in addition to the sieving mechanism, the surface charge of the support layer allows rejecting charged ions with a size smaller than the membrane pore size. These characteristics enable NF to retain multivalent ions while allowing the passage of small uncharged species and monovalent ions. This aspect, combined with low energy consumption compared to reverse osmosis, makes NF promising for the rejection of heavy metals from wastewater [13,14]. For the treatment of complex wastewaters, NF has been investigated as a standalone process [15,16,17,18] as well as in integration with other treatment methods such as coagulation and ultrafiltration [19,20].

In the current study, NF has been applied for the treatment of wastewater streams from a Danish anodizing industry according to point source (the treatment of individual streams at their point of origin) and end-of-pipe (the treatment of mixed streams at a centralized location) treatment strategies. In the first scenario, various individual wastewater streams, including color rinse, alkaline pickling rinse, acidic pickling rinse and anodizing rinse, have been treated separately through NF. According to the second protocol, all streams have been collected to make a mixed wastewater stream before treatment. Based upon the rejection of various ions and the process stability, the optimum treatment scenario has been identified. The study has been carried out by using a commercial NF99HF membrane, due to its reported high flux in desalination and wastewater treatment applications [21].

## 2. Materials and Methods 

### 2.1. Wastewater Analysis

**Figure 1:**
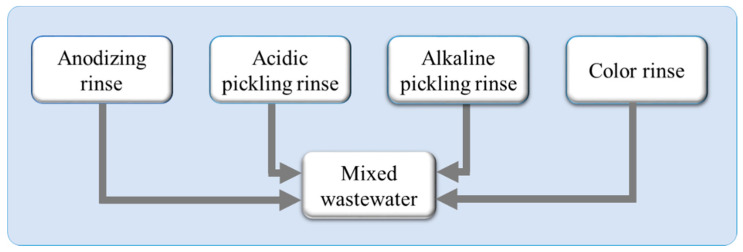
Overview of the different solutions treated with nanofiltration (NF).

Wastewater was collected from four rinse baths: color rinse, alkaline pickling rise, acidic pickling rinse and anodizing rinse, as well as from a centralized container where wastewater from the individual baths was mixed (Figure 1) and neutralized. The collected wastewater streams were characterized in terms of ionic composition, water activity, pH, conductivity and dry matter content. The ionic compositions were measured with an inductively coupled plasma spectrometer (ICP) (iCap 6300 DUO; Thermo Scientific, Waltham, MA, USA). The samples were measured in radial view. A total of 1 ppm Yttrium was used as the internal standard. All ICP measurements were made in duplicates. Water activity was measured at 25 °C by the dew point method using a chilled mirror (Aqualab 4TE). Dry matter was determined by the weight loss using 10 mL of sample for 24 h at 105 °C. The absorbance of the color rinse solution was measured by UV-Vis spectrophotometer (Thermo Fisher scientific) at 660 nm. 

The measured characteristics of the wastewater streams are listed in Table 1. Due to the extreme pH of the alkaline pickling rinse, acidic pickling rinse and the anodizing rinse, it was necessary to adjust pH to bring it within the safe operating range of NF membrane (defined by the manufacture). Potentiometric titrations were performed using Titralab™ 900 equipment from Radiometer, Brønshøj, Denmark, using either 2M H_2_SO_4_ or 2M NaOH as titrant. pH was measured using an SI Analytics Blue Line 17 pH glass electrode calibrated against standard buffer solution at pH 4 and 7. Depending on pH, the wastewater was adjusted with H_2_SO_4_ or NaOH up to pH 4 or down to pH 9.5 (safe operating window of the applied membrane) and prior to nanofiltration, the removal of precipitants was performed through centrifugation (Thermo Scientific) after pH adjustment. 

### 2.2. NF Tests

**Figure 2:**
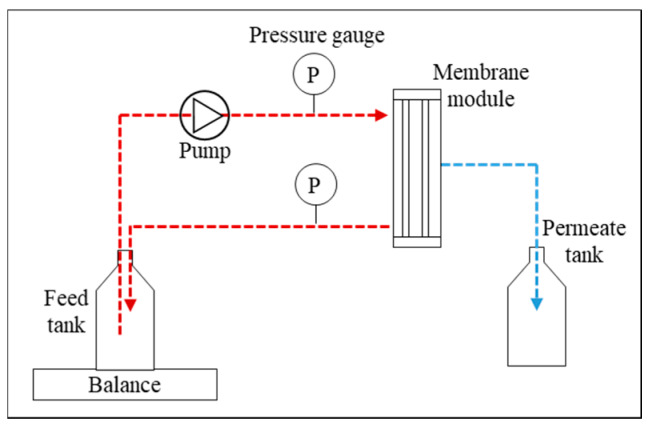
Schematic overview of the NF system.

NF tests on all wastewater streams were carried out at a low pressure of 3.5 bar by using a setup containing an Alfa Laval LabStak M10 crossflow module (Figure 2) with a crossflow pump (ZUWA, Nirostar, Austria) as well as two pressure gauges (Danfoss, Pressure Transmitter). The effective membrane area of the M10 module was 0.0168 m^2^. The crossflow was 1 L/min. The membranes were the Alfa Laval-NF99HF flat sheet thin film composite membrane with a pore radius around 0.43 nm according to Oatley et al. [22] and a recommended pH range of 3 to 10 [23]. New membranes were used for each experiment and each membrane was activated according to the procedure provided by the manufacturer. First, the membranes were rinsed with distillate water and then a warm (30–55 °C) solution of distillate water was introduced into the system. Afterwards, the membranes were rinsed with a solution of NaOH (pH 8.5–10.5) for 30 min and finally rinsed with distillate water until neutral pH. Hereafter, the different feed solutions were introduced into the system with an initial volume of 3 L for all the experiments. 

Rejections were calculated from Equation (1). The concentration of ions in permeate and retentate were measured several times during the experiment.

(1) $$R=1-\frac{C_{p}}{C_{r}}$$

where *C_p_* and *C_r_* are the concentration in permeate and retentate, respectively.

## 3. Results and Discussion

### 3.1. Effect of pH Adjustment

**Figure 3:**
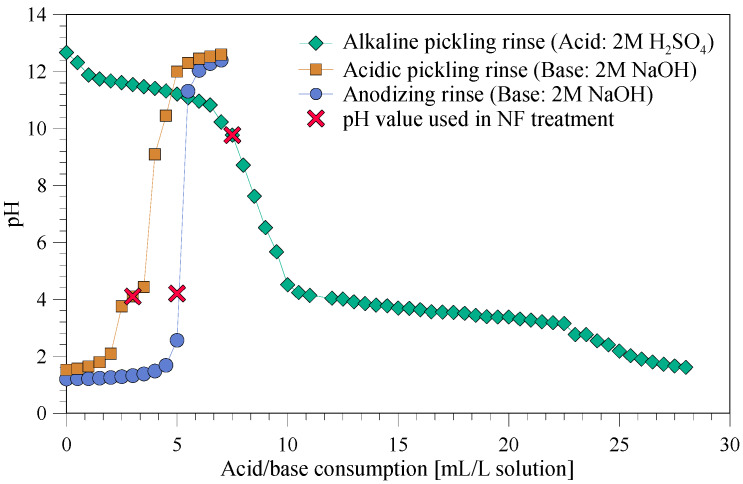
Titration curve for some of the wastewater samples.

Prior to NF, pH was adjusted to 9.5, 4.2 and 4.0 for the alkaline pickling rinse, acidic pickling rinse and the anodizing rinse, respectively. The titration curve for the alkaline pickling rinse, acidic pickling rinse and the anodizing rinse, showed the required amount of NaOH or H_2_SO_4_ for the pH adjustment (Figure 3).

As a result of pH adjustment, precipitate was formed and removed through centrifugation. For the alkaline pickling rinse, the dry matter content (total dissolved solid content) was thereby reduced from 6% to 3.6%, i.e., 24 g of dry material was removed per kg of wastewater (Table 1 and Table 2). As a result, water activity increased from 0.98 to 0.99 (Table 1 and Table 2). The ICP results for the alkaline pickling rinse were also measured before and after pH adjustment and showed that all heavy metals, e.g., Cr, Cu and Mn, were precipitated and removed (Table 1 and Table 2). The concentration of Al and P in effluent was reduced with 99.5%–99.7%, and the Al accounts for 40% of the dry material removed. Assuming that all Al is precipitated as AlOOH, it results in 20 g of dry material per kg of wastewater, and, assuming that all Al precipitate as Al(OH)_3,_ it results in 26 g per kg of wastewater, which agrees well with the measured dry matter content before and after pH adjustment. Na concentration declined due to dilution during pH adjustment. The measured buffer capacity at pH 11.5 may be due to precipitation (Figure 3). Dissolved aluminium exists as Al(OH)_4_^−^ at high pH and has to be neutralized if aluminium is precipitated, i.e., 1 mole of H_2_SO_4_ is required to neutralize 2 moles of Al(OH)_4_^−^. The total concentration of aluminium prior to pH adjustment was 0.33 M.

For acidic pickling rinse, the dry matter content of the sample increased from 0% to 1.6% after the addition of NaOH, meanwhile the activity remained almost the same. Comparing the ICP results before and after pH adjustment showed that the concentration of ions decreased marginally, whereas Na increased by more than 70 times due to the addition of NaOH. Precipitate was formed after pH adjustment and after centrifugation as 15% of the iron was removed (0.7 ppm) and 6% of the aluminum was removed (3.3 ppm). The precipitation of iron or aluminum may also explain the buffer effect around pH 4. For the anodizing rinse, NaOH addition increased the dry matter content from 0.7 to 1.1%, whereas the conductivity dropped from 24.1 to 10 mS/cm. The higher dry matter content was, again, an effect of the addition of NaOH, and the reduced conductivity, which may largely be an effect of the lower concentration of H^+^ after pH adjustment. The conductivity of hydrochloric acid was 26 S cm^−1^ at pH 1.2 assuming a molar conductance of 400 S mol^−1^ cm^2^. There were no significant changes to other ions except Na. The mixed wastewater already contained large amounts of precipitate due to the pH neutralization of the wastewater. The composition of the mixed wastewater was measured both before (W—Table 1) and after centrifugation (W*—Table 2). Neither Cr, Cu, Fe, Ni, P, Pb nor Zn were detected in the mixed wastewaters (W and W*), as the company neutralizes pH and remove the ions as precipitates. 

### 3.2. Nanofiltration of Various Wastewaters

It was possible to remove contaminants with pH adjustment, but it was not possible to remove all critical heavy metals; thus, NF was tested as an alternative or supplementary method. All the baths were treated through NF in order to find the optimum scenario for wastewater treatment. For mixed wastewater, two tests were performed, i.e., one where the wastewater was used as such and another test, where it was centrifuged prior to use.

The water permeability of the membrane was measured to 11 L/(m^2^·h·bar). Other studies of the permeability for these membranes are in the range of 9–18 L/(m^2^·h·bar) [24]. Thus, the permeability in this study is in the lower end of the range, but still reasonable.

**Figure 4:**
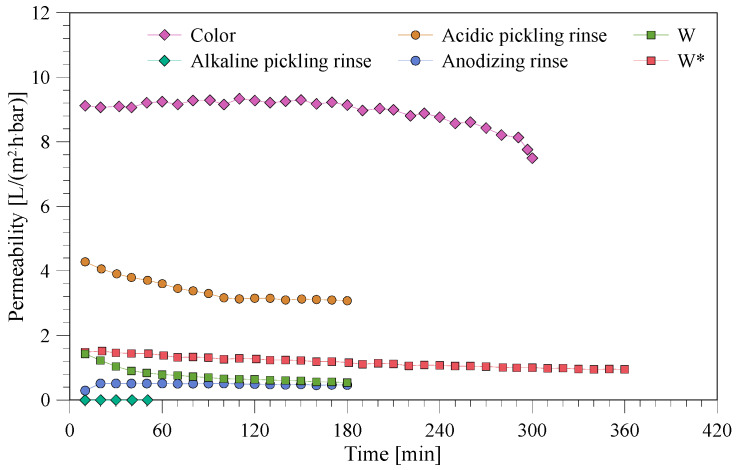
Permeability during the NF treatment of different baths from the anodizing.

The permeability values for all the different bath experiments are shown in Figure 4. The experimental time was prolonged for the baths, which showed promising results in terms of permeability and rejections (color rinse and mixed wastewater after centrifugation). The permeability for NF of the color rinse was measured to be 9 L/(m^2^·h·bar) but started to drop at the end of the experiment. The achieved recovery factor (RF) of the color rinse was 88.1%. The permeability was higher than the other wastewater streams, which corresponds to the low conductivity of the color rinse (~0.06 mS/cm). The NF of acidic pickling rinse showed a declining permeability that ends at 3.2 L/(m^2^·h·bar) and an RF of 31.4%. The NF of the mixed wastewater showed a stable permeability around 1.5 L/(m^2^·h·bar), with an RF of 13.9% if the precipitate was removed prior to filtration. For the three other baths (alkaline pickling rinse, anodizing rinse and the mixed wastewater without centrifugation), the permeability was lower than 0.5 L/(m^2^·h·bar) and these baths are not efficiently treated since they have an RF value below 4.5%. In particular, for the alkaline pickling rinse, no permeate was observed at 3.5 bar. 

**Figure 5:**
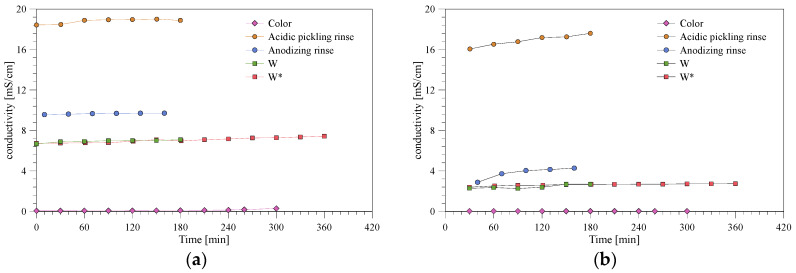
Conductivity of (**a**) feed and (**b**) permeate for the different baths.

The conductivity of the feed solution and permeate was measured during the experiment (Figure 5). The conductivity of the color rinse was lower compared to the other baths and the feed conductivity increased from 0.06 to 0.29 mS/cm, which corresponds to an ion rejection between 67% and 92% during the experiment. The conductivity of the permeate was 0.02 mS/cm. The rejection of ions, when filtering the acidic pickling rinse, was low and measured to 7% to 13%. The used membrane has an isoelectric point between 4.12–4.42 [22]. The treatment of the acidic pickling rinse was carried out in the range of the isoelectric point, i.e., at pH 4.2; thus, below this pH, the membrane is positively charged. The reason for the low rejection, measured through the conductivity, may be due to the high concentration of both NO_3_^−^ and Na^+^ (3858 and 3139 ppm—Table 1 and Table 2), which is easily transported through the neutrally charged membrane, as both nitrate and sodium ions are well below the pore size of the membrane. The anodizing rinse showed an ion rejection of 80% in the beginning of the experiment, which drops to 58% due to the increase in the conductivity in the permeate. The ionic strength increases when ions are concentrated in the feed in which the electric double layer is compressed, e.g., in the pores, which may explain the lower rejection of ions (Figure 5b). The mixed wastewater solution both before and after centrifugation showed a stable ion rejection of around 64% during the experiments. 

**Figure 6:**
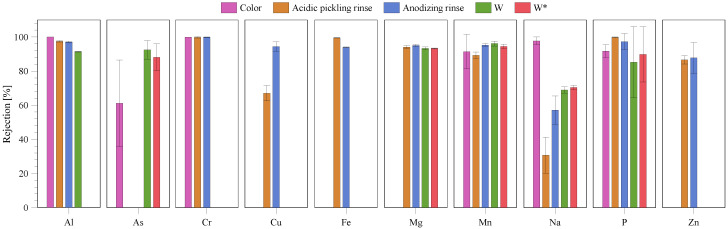
Rejection of each ion for the different baths. Rejection is not shown for some baths where the concentration of ions is below the detection limit in both the feed and permeate.

An ICP was used to determine the concentration of the ions in feed and permeate. From the ICP data, the rejection was calculated for each ion (Figure 6). The rejection is the average rejection of each ion during the experiment. For the acidic pickling rinse, the rejection of Na was 31% and the rejection of Cu was 67%, while the rejection of the other ions ranged from 80% to 100%. The NF of anodizing rinse, mixed wastewater without centrifugation and the mixed wastewater with centrifugation showed high rejections even for Na (57%, 69% and 70%, respectively). For mixed wastewater, both with and without centrifugation, the rejection of Mg, Mn and As was higher than 80% and none of the heavy metals were detected in the permeate.

**Figure 7:**
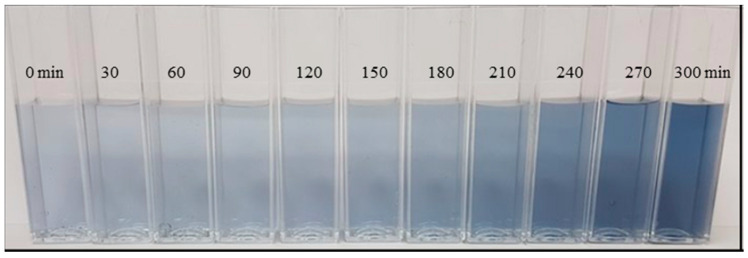
Change in concentration of the color rinse bath during filtration.

**Figure 8:**
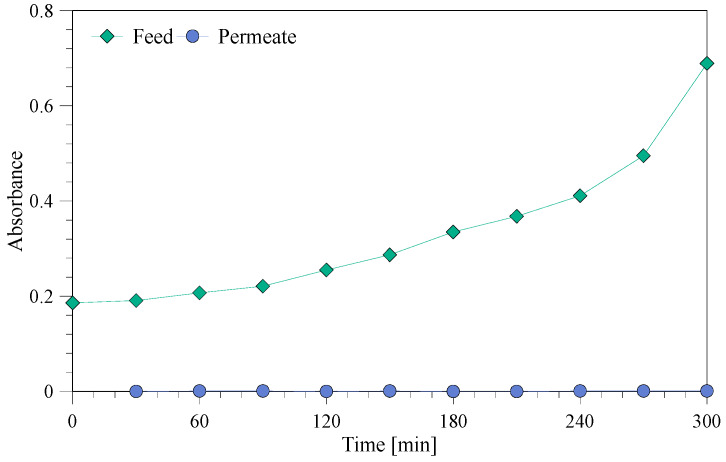
Absorbance during treatment of the color rinse (measured at 610 nm).

To test if the membrane rejected color from the color rinse bath, the color change was observed during the experiment both visually (Figure 7) and through absorbance (Figure 8), which proved that no color was transported through the membrane. The concentrated color rinse can potentially be reused as a new color bath. 

### 3.3. Permeate Quality

**Figure 9:**
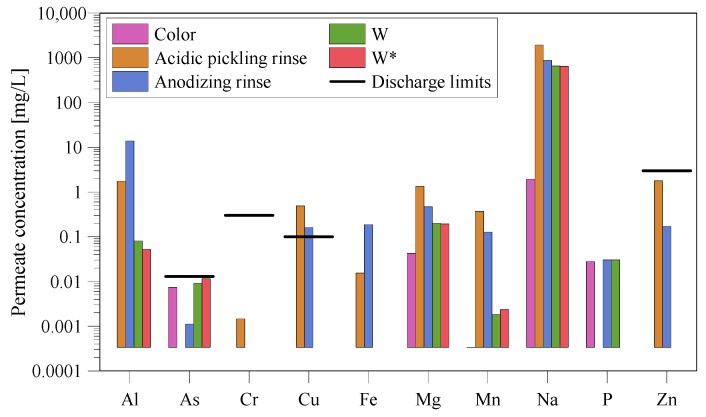
Permeate quality for the different baths.

The permeate quality was tested by measuring the final ion concentration in the permeate stream (Figure 9). The discharge limit given by Danish standards [25] is illustrated as a black line, and it is seen that only Cu concentrations, in the acidic pickling rinse and in the anodizing rinse, are exceeding the maximum concentration. Additionally, the permeate quality meets the criteria for discharge. For water reuse, other criteria may be relevant depending on the industry. 

## 4. Conclusions

Nanofiltration was used for both point source and the end-of-the-pipe treatment of wastewater from the anodizing industry. pH adjustment was required for the alkaline, acidic and anodizing waste streams to avoid damaging the membrane. For the alkaline stream, pH adjustment resulted in the precipitation of dry matter, including heavy metals, in a significant quantity (40%). However, the alkaline stream could not be filtered through the NF membrane. For wastewater from the color rinse bath, NF showed stable flux and good-quality permeate with a low concentration of heavy metals and very low electrical conductivity. The acidic pickling rinse showed a slight decrease in NF permeability over time and a high rejection of cations; however, the concentration of Cu remained above the recommended discharge limit. NF exhibited stable flux for anodizing rinse and showed high rejection towards various ions but also with a Cu concentration slightly higher than the allowable discharge limit. No heavy metals were detected in NF permeate of the neutralized mixed wastewater. Furthermore, the flux remained stable for the mixed wastewater if large particles were removed before NF. Therefore, end-of-the-pipe treatment appears to be more promising than point source treatment for discharge purposes. For water reuse, the treatment of the color rinse and anodizing rinse baths may be a better solution, because permeate with low conductivity can be produced. Thus, the final treatment strategy should be adopted according to the final aim, i.e., reuse or discharge.

## Figures and Tables

**Table 1:** Composition and characteristics of the different wastewater solutions.

	Color Rinse	Alkaline Pickling Rinse	Acidic Pickling Rinse	Anodizing Rinse	Mixed Wastewater (W)
pH	7.4	12.7	1.2	1.52	7.5
Conductivity [mS/cm]	0.06	35.9	76.1	24.1	6.7
Dry matter [%]	0	6.0	0	0.7	0.7
Water activity	N.A.	0.9837	0.9964	0.9992	0.9977
Al [ppm]	0.0838	9040.6	56.78	510.84	0.678
As [ppm]	0.149	N.D	N.D	N.D	0.093
Cr [ppm]	0.962	2.36	0.56	0.38	N.D
Cu [ppm]	N.D	0.36	1.33	2.05	N.D
Fe [ppm]	N.D	3.35	5.06	2.95	N.D
Mg [ppm]	N.D	1.31	18.43	8.62	2.74
Mn [ppm]	0.0108	2.23	2.82	2.39	0.092
Na [ppm]	11.19	4275.7	43.07	20.65	N.A
Ni [ppm]	N.D	N.D	4.69	N.D	N.D
P [ppm]	0.183	46.30	0.48	0.63	N.D
Pb [ppm]	N.D	N.D	N.D	N.D	N.D
Zn [ppm]	N.D	0.24	10.51	1.34	N.D.
Cl [ppm]	8	623.6	33.78	5.68	N.A
NO [ppm]	N.D	N.D	9.66	N.D	N.A
NO_3_ [ppm]	N.D	150.8	3858	36.7	N.A
SO_4_ [ppm]	37.64	64.2	16.1	3570	N.A

N.A: Not analyzed, N.D: Below detection limit.

**Table 2:** Characteristics after pH adjustment and for the mixed wastewater after centrifugation.

	Alkaline Pickling Rinse ^a^	Acidic Pickling Rinse ^a^	Anodizing Rinse ^a^	Mixed Wastewater (W*) ^b^
pH	9.5	4.2	4.0	7.5
Conductivity [mS/cm]	36	18	10	7
Dry matter [%]	3.6	1.6	1.1	0.5
Water activity	0.9924	0.9959	0.9991	0.9993
Al [ppm]	37.11	50.02	488.34	N.D
As [ppm]	N.D.	N.D.	N.D.	0.022
Cr [ppm]	N.D.	0.48	0.37	N.D
Cu [ppm]	N.D.	1.22	1.99	N.D
Fe [ppm]	0.021	4.02	2.93	N.D
Mg [ppm]	N.D.	16.97	8.60	2.68
Mn [ppm]	N.D.	2.61	2.40	0.035
Na [ppm]	4205.6	3138.6	1882.1	2016.0
Ni [ppm]	N.D.	N.D.	N.D.	N.D
P [ppm]	0.16	3.7	0.65	0.068
Pb [ppm]	N.D.	N.D.	N.D.	N.D
Zn [ppm]	N.D.	10.06	1.31	N.D

^a^ after pH adjustment, ^b^ after centrifugation, N.A: Not analyzed, N.D: Not detected.
